# Curcumin, demethoxycurcumin, and bisdemethoxycurcumin induced caspase-dependent and –independent apoptosis via Smad or Akt signaling pathways in HOS cells

**DOI:** 10.1186/s12906-020-2857-1

**Published:** 2020-03-03

**Authors:** Cheng Huang, Hsu-Feng Lu, Yu-Hsuan Chen, Jui-Chieh Chen, Wen-Hsiang Chou, Hsiu-Chen Huang

**Affiliations:** 1 Department of Biotechnology and Laboratory Science in Medicine, National Yang-Ming University, Taipei, 11221 Taiwan; 2 Department of Earth and Life Sciences, University of Taipei, Taipei, 11153 Taiwan; 3 Departments of Clinical Pathology, Cheng Hsin General Hospital, Taipei, 11221 Taiwan; 4 Department of Restaurant, Hotel and Institutional Management, Fu-Jen Catholic University, New Taipei, 24205 Taiwan; 5 Department of Applied Science, National Tsing Hua University South Campus, No.521, Nanda Rd, Hsinchu City, 30014 Taiwan; 6 Department of Biochemical Science and Technology, National Chiayi University, Chiayi, 60004 Taiwan; 7 School of Medicine, National Defense Medical Center, Taipei, 11490 Taiwan; 8 Department of Orthopedics, Cheng Hsin General Hospital, Taipei, 11220 Taiwan

**Keywords:** Curcumin, Demethoxycurcumin, Bisdemethoxycurcumin, Apoptosis, Osteosarcoma

## Abstract

**Background:**

Osteosarcoma is the most common primary malignant bone tumor in children and adolescents and has also been associated with a high degree of malignancy and enhanced metastatic capacity. Curcumin (CUR) is well known for its anti-osteosarcoma activity. However, both demethoxycurcumin (DMC), and bisdemethoxycurcumin (BDMC) are natural curcumin analogues/congeners from turmeric whose role in osteosarcoma development remains unknown.

**Methods:**

To evaluate the growth inhibitory effects of CUR, DMC and BDMC on osteosarcoma (HOS and U2OS), breast (MDA-MB-231), and melanoma (A2058) cancer cells, we employed the MTT assay, annexin V-FITC /7-AAD staining, and clonogenic assay.

**Results:**

CUR,DMC, and BDMC all decreased the viability of HOS, U2OS, MDA-MB-231, and A2058 cancer cells. Additionally, CUR,DMC, and BDMC induced the apoptosis of HOS cells through activation of Smad 2/3 or repression of Akt signaling pathway. Furthermore, the combination of CUR,DMC, and BDMC synergistically reduced cell viability, colony formation and increased apoptosis than either two or a single agent in HOS cells.

**Conclusions:**

The combination of these three compounds could be used as a novel target for the treatment of osteosarcoma.

## Background

Turmeric is a yellow Indian spice obtained from the rhizome of *Curcuma longa* Linn which is a type of herb belonging to Zingiberaceae family. The three major chemical constituents and biological activities of turmeric were approximately 77% curcumin (CUR), 17% demethoxycurcumin (DMC), and 3% bisdemethoxycurcumin (BDMC), which are belong to curcuminoids [1, 2]. Cur, a linear diarylheptanoid, is a natural phenolic compound and it has been well known and used to treat many diseases, such as cancer [3–6], diabetes [7, 8], inflammation [1] and neurodegenerative disorders [9, 10]. In spite of the potential health benefits of CUR, its clinical applications are limited due to its low oral bioavailability, low water solubility, and instability at acidic Ph [11, 12]. DMC and BDMC are naturally occurring CUR analogues, which lacks one or two methoxy groups on the aromatic rings of CUR [13]. DMC and BDMC exhibited much better chemical stability than CUR and both have been recently reported to exhibit anticancer properties [14]. Until now, the detailed mechanisms of DMC and BDMC in cancer prevention are still mostly unknown, and these two compounds must remain to be investigated.

Osteosarcoma is one of the most common primary malignant bone tumor originating from mesenchymal stem cells, and it occurred predominantly in late childhood and early adolescence [15]. Chemotherapy in treating nonmetastatic osteosarcoma patients has dramatically improved the five-year survival rates from < 20 to 60%–70% [16–18]. While osteosarcoma is not very sensitive to chemotherapy [19, 20]. Thus, it is difficult to develop more effective treatments. Recently, curcuminoids have been increasingly used for the treatment of osteosarcoma to overcome chemoresistance and increased the apoptotic rate of osteosarcoma cells [21]. Apoptosis, or programmed cell death (PCD), is a regulated cellular suicide and the inducing apoptosis plays a critical role in the development of new anticancer drugs [22]. CUR, the principal curcuminoid of turmeric, has also been reported to induce apoptosis in various cancer cells, and have the potential for being developed as cancer therapeutic agents. Although CUR also has shown to induce cell death and cell cycle arrest in HOS cells, the mechanism has not been fully established [23]. Whether analogs of CUR, such as DMC and BDMC also could regulate osteosarcoma cell proliferation to a similar extent as CUR and the mechanisms remain to be investigated. To our knowledge, this is the first time study to identify the anti-cancer effects of DMC and BDMC on osteosarcoma cells. Thus, we will investigate the effects of CUR, DMC and BDMC on cell proliferation in osteosarcoma (HOS and U2OS), breast (MDA-MB-231), and melanoma (A2058) cancer cells in the present study.

## Methods

### Chemicals

Curcumin (CAS Number 458-37-7, Sigma-Aldrich, Purity: ≧99.5%), demethoxycurcumin (CAS Number: 22608-11-3, Sigma-Aldrich, Purity: ≧98%), bisdemethoxycurcumin (CAS Number: 33171-05-0, Sigma-Aldrich, Purity: ≧98%), Thiazolyl Blue Tetrazolium Bromide (MTT) were purchased from Sigma–Aldrich (CAS Number: 298-93-1). β-actin (Cat No. GTX109639), p-p38 (Cat No. GTX24822), p-Akt (Cat No. GTX128414), p-Erk (Cat No. GTX24819), p-smad2 (Cat No. GTX133475), p-smad3 (Cat No. GTX129841), cleaved PARP (Cat No. GTX132329), cleaved caspase 3 (Cat No. GTX86952) antibodies were purchased from GeneTex. Annexin V Alexa Fluor 488 Ready Flow Conjugate **(**Lot number 2081235) and 7-aminoactinomycin D (7-AAD, **Catalog number:** A1310) both were purchased from Thermo Fisher Scientific.

### Cell line and cell culture

The U2OS, HOS, A2058, MDA-MB-231 cell lines were obtained from Bioresource Collection and Research Center (BCRC, Hsinchu, Taiwan). Cells in culture were not more than 10 passages from the time of receipt. Cells were cultured in Dulbecco’s Modified Eagle’s Medium (DMEM) supplemented with antibiotics (100 U/mL of penicillin A and 100 U/mL of streptomycin) and 10% fetal bovine serum (FBS), and maintained at 37 °C in 5% CO_2_ humidified air.

### Cell viability assay

The effect of CUR, DMC and BDMC on cell viability in U2OS, HOS, A2058, and MDA-MB-231 cells were examined by MTT assay. Briefly, cells were seeded into 12-well plates with 10% FBS overnight. After attachment, the cells were put into 2% FBS media and then treated with varying concentrations of CUR, DMC and BDMC alone or in combination. After incubation for 24–48 h, 300 μL of MTT working solution (0.5 mg/mL) was added to each well and incubated for 3 h at 37 °C. The supernatant was aspirated, and the MTT-formazan crystals formed by metabolically viable cells were dissolved in DMSO. Finally, the absorbance was monitored by a 96-well microplate reader at a wavelength of 540 nm.

### Flow cytometric analysis of apoptosis

HOS cells were cultured at a density of 1 × 10^6^ cells in 10-cm dishes with 10*%* FBS overnight. After attachment*,* the cells were put into 2*%* FBS media and then treated with varying concentrations of CUR, DMC and BDMC alone or in combination. After treatment with varying concentrations of CUR, DMC and BDMC for 24 h, we collected the cell pellet by trypsinization and centrifugation at 2000 g for 5 min, and then discard the supernatant. Finally*,* 500 μL of 1 × annexin V-FITC Binding Buffer was added to resuspend the pellet, and then 5 μl of annexin V-FITC and 5 μL of 7-AAD were added to the cells. The samples were gently vortexed and incubated 15 min at 4 °C in the dark. The Flow cytometric analysis was performed by FAC-Scan cytometry (BD Biosciences, San Jose, CA).

### Nuclear morphology assay and immunofluorescence assay

Nuclear morphology was detected by Hoechst staining. Immunofluorescence study was detected by apoptosis-inducing factor (AIF) primary antibodies. The HOS, U2OS, and A2058 cells were cultured with 10*%* FBS on coverslips placed in six-well plates for overnight. After attachment*,* the cells were changed into 2*%* FBS media and then treated with 10–20 μM of CUR, DMC and BDMC. After treatment with CUR, DMC and BDMC for 24 h, HOS, U2OS, and A2058 cells were washed with phosphate buffered saline (PBS), and then fixed with 4% formaldehyde in PBS for 30 min at room temperature. Finally, HOS cells were added apoptosis-inducing factor (AIF) primary antibodies at 4 °C overnight, followed with the fluorescein isothiocyanate-conjugated secondary antibodies for 1 h, and then were added 4 mg/mL Hoechst 33258 for 30 min, but U2OS, and A2058 cells were only added 4 mg/mL Hoechst 33258 for 30 min. The coverslips were mounted in Vectashield mounting medium and the nuclear morphology and AIF nuclear translocation were viewed under confocal laser scanning microscopy.

### Western blot analysis

HOS cells with or without 10–20 μM CUR, DMC and BDMC treatment for 24 h were washed with PBS and lysed in the Golden lysis buffer containing protease inhibitors for 30 min at 4 °C. Protein content was determined against a standardized control, using the Bio-Rad protein assay kit (Bio-Rad Laboratories). The protein inputs in the western blot analyses were normalized by loading equal amounts of total protein lysates into the SDS-PAGE gel. Transferred onto polyvinylidene difluoride membranes, and then probed with primary antibody (β-actin, p-p38, p-Akt, p-Erk, p-Smad2, p-Smad3, cleaved PARP, or cleaved caspase 3) followed by secondary antibody conjugated with horseradish peroxidase. Reactive bands were visualized with enhanced chemiluminescence (ECL) reagents.

### Colony formation assay (Clonogenic assay)

Cell proliferation was monitored using a colony formation assay. Briefly, HOS cells were seeded into 24-well plates with 10% FBS overnight. After attachment, cells were maintained in DMEM containing 10% FBS with or without various concentrations of CUR, DMC and BDMC for an additional 14 days. At last, the cells were fixed with 4% paraformaldehyde for 15 min at room temperature and stained with crystal violet dye for 5 min.

### Statistical analysis

All values were expressed as mean ± SD. Each value is the mean of at least three separate experiments in each group. Student’s t-test was used for statistical comparison. * indicates that the values are significantly different from the control (*, *p* < 0.05; **, *p* < 0.01; ***, *p* < 0.001).

## Results

### CUR, DMC and BDMC inhibited the cell viability of HOS, U2OS, MDA-MB-231, and A2058 cells

**Fig. 1:**
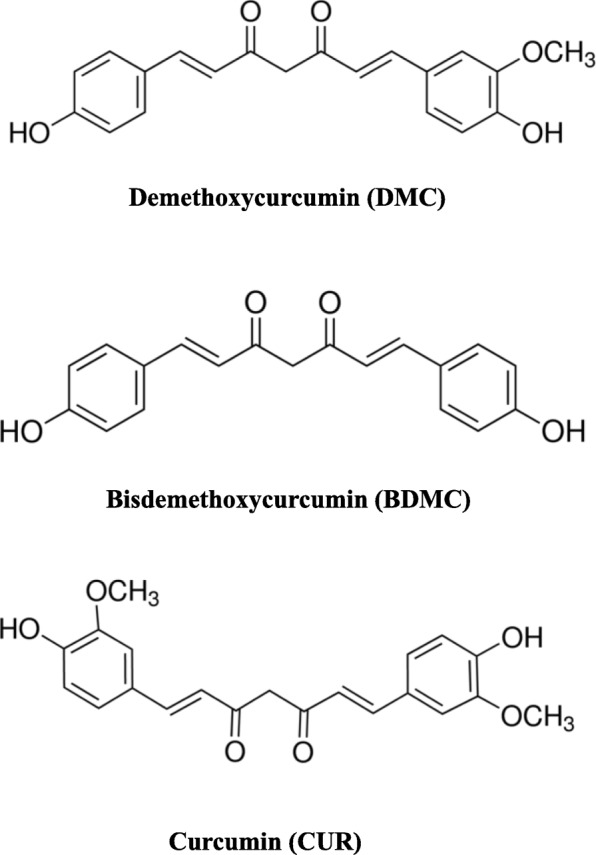
The chemical structure of Curcumin (CUR), demethoxycurcumin (DMC), and bisdemethoxycurcumin (BDMC)

**Fig. 2:**
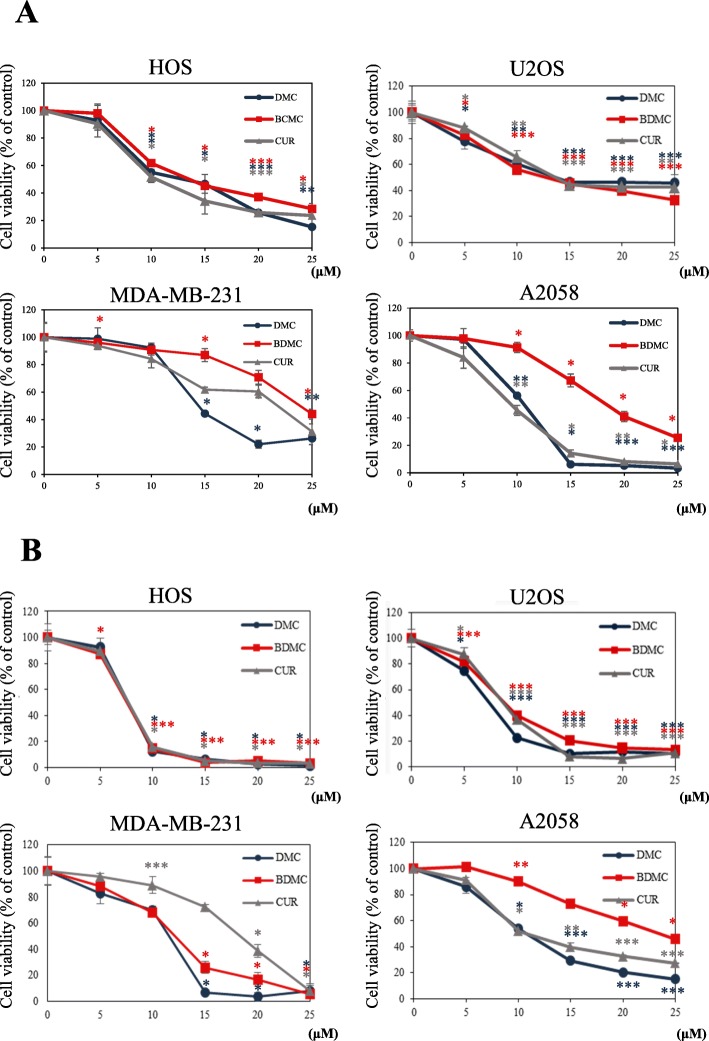
Effect of cell viability of CUR, DMC, and BDMC in HOS, U2OS, MDA-MB-231, and A2058 cancer cells. The cells were treated with 5–25 μΜ CUR, DMC and BDMC for (**a**) 24 and (**b**) 48 h, and cell viability was measured by the MTT assay. The percentage of cell viability was calculated as the ratio: 100% x (A540 absorbance of the drug-treated wells / (A540 absorbance of the no drug-treated wells). * Indicates the values are significantly different than the control (*P < 0.05, **P < 0.01, ***P < 0.001)

To evaluate the growth inhibitory effects of CUR, DMC and BDMC (Fig. 1) on osteosarcoma (HOS and U2OS), breast (MDA-MB-231), and melanoma (A2058) cancer cells, we employed the MTT assay. Treatment with 5–25 μM of CUR, DMC and BDMC for 24–48 h significantly resulted in inhibition of the HOS, U2OS, MDA-MB-231, and A2058 cell viability in a time- and concentration-dependent manner, but to varying extents (Fig. 2a and b). Osteosarcoma (HOS and U2OS) cells were more sensitive to CUR, DMC and BDMC than breast (MDA-MB-231) and melanoma (A2058) cancer cells. Thus, the HOS and U2OS cell lines were selected for further analysis. Treatment of CUR, DMC and BDMC for 24–48 h at the same concentration had the same inhibiting effect on HOS and U2OS cells. However, treatment of CUR and DMC for 48 h were more active than BDMC in inhibiting A2058 cell growth. Treatment of BDMC and DMC for 48 h were more active than CUR in inhibiting MDA-MB-231 cell growth.

### CUR, DMC and BDMC induced apoptosis in HOS cells

**Fig. 3:**
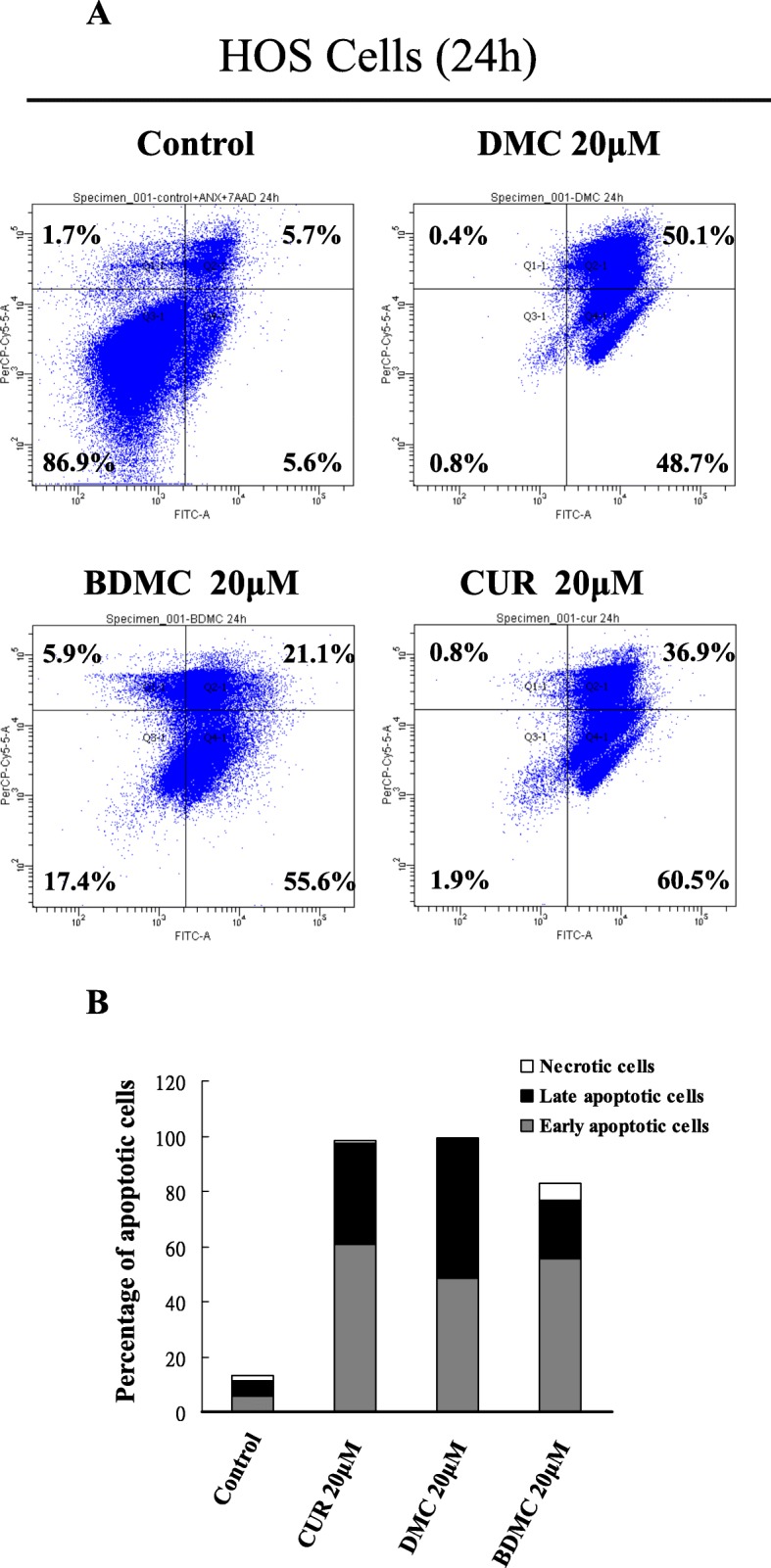
Effects of CUR, DMC, and BDMC on the apoptosis of HOS cells. **a** HOS cells were treated with 20 μM CUR, DMC, and BDMC for 24 h and stained with annexin V-FITC /7-AAD, and analyzed by flow cytometry. **b** Quantitative analysis of the percentage of early apoptotic and late apoptotic cells in HOS cells

**Fig. 4:**
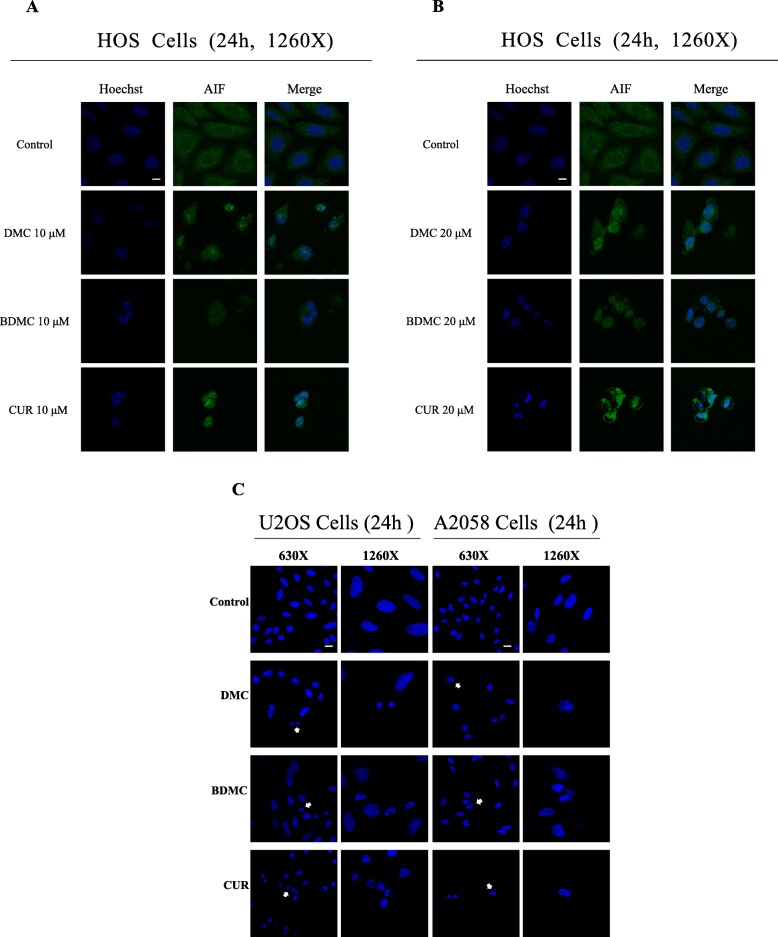
Effects of CUR, DMC, and BDMC on nuclear morphology or AIF nuclear translocation in HOS, U2OS, and A2058 cancer cells. HOS cells treated with (**a**)10 μΜ and (**b**) 20 μΜ CUR, DMC, and BDMC for 24 h and stained with Hoechst 33258 (blue) and AIF antibody (green) and viewed under confocal laser scanning microscopy. The bar in the images represents 5 μm in length. **c** U2OS and A2058 cancer cells treated with 20 μΜ CUR, DMC, and BDMC for 24 h and stained with Hoechst 33258 (blue) and viewed under confocal laser scanning microscopy. The bar in the images represents 10 μm in length.

To identify whether CUR, DMC and BDMC could increase apoptosis in HOS cells, we used the annexin V-FITC and 7-ADD double staining assay. Cells were treated with CUR, DMC and BDMC (20 μM) for 24 h, and stained with annexin V-FITC /7-AAD, and analyzed by flow cytometry. Early apoptotic cells were positive for annexin V staining and negative for 7-ADD staining, necrotic cells were for negative annexin V staining and positive for 7-ADD staining, whereas late apoptotic cells were positive for both annexin V-FITC and 7-ADD staining. The percentage of early apoptotic cells increased from 5.6 to 60.5%, 48.7 and 55.6% after treatment with CUR, DMC and BDMC for 24 h, respectively. The percentage of necrotic cells increased from 1.7 to 0.8%, 0.4 and 5.9% after treatment with CUR, DMC and BDMC for 24 h, respectively. The percentage of late apoptotic cells were increased from 5.7 to 36.9%, 50.1 and 21.1% after treatment with CUR, DMC and BDMC, respectively (Fig. 3a and b). Thus, CUR, DMC and BDMC significantly increased apoptosis (including early and late apoptosis) in HOS cells. Typical morphological characteristics of apoptotic cells could be observed through using fluorescence microscopy by nuclear staining with Hoechst 33342. Next, we would provide direct evidence of CUR, DMC and BDMC -induced apoptosis in HOS, U2OS, and A2058 cells by using Hoechst day to observe the nuclear morphology. As shown in Fig. 4a, b and c, we observed increased numbers of apoptotic cells in HOS, U2OS, and A2058 cells treated with CUR, DMC and BDMC for 24 h. AIF nuclear translocation was a critical mediator of caspase-independent cell death. To determine whether the CUR, DMC and BDMC (10–20 μM) induced caspase-independent cell death, the localization of AIF in HOS cells was observed using immunofluorescence staining experiments. As shown in Fig. 4a and b, AIF nuclear translocation was significantly increased in HOS cells treated with 10–20 μM CUR, DMC and BDMC for 24 h.

### CUR, DMC and BDMC induced caspase 3 and PARP activation and increased Smad2/3 phosphorylation in HOS cells

**Fig. 5:**
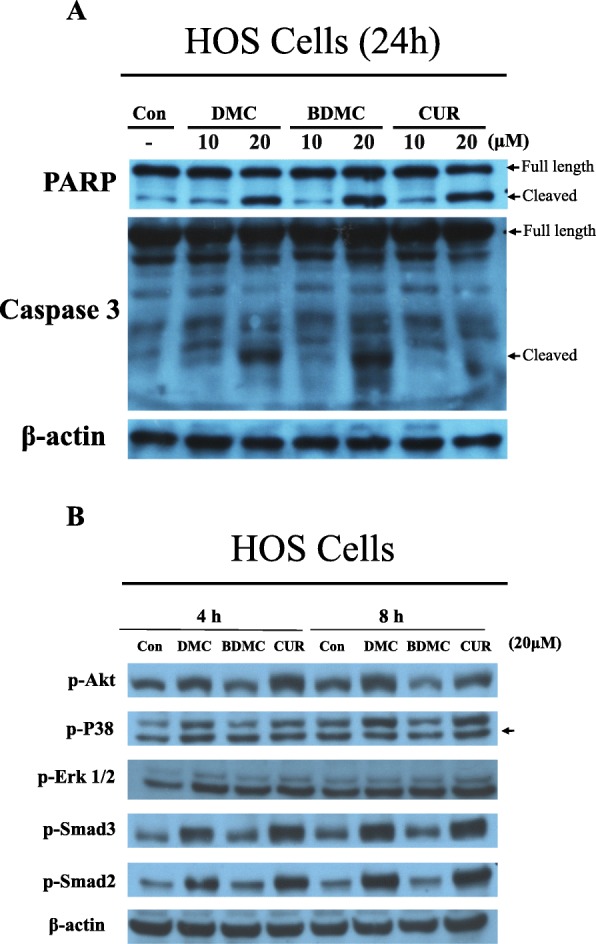
**a** The expression of full length caspase-3, cleaved caspase-3, full length PARP and cleaved PARP in HOS cells treated with 10–20 μΜ CUR, DMC, and BDMC for 24 h were detected using the western blotting assay. **b** Expression of p-Akt, p-p38, p-Erk 1/2, p-Smad2 and p-Smad 3 in HOS cells treated with 20 μΜ CUR, DMC, and BDMC for 4–8 h was detected using the western blotting assay

In order to identify the molecular mechanism for CUR, DMC and BDMC -induced apoptosis, western blot analysis was carried out to profile the expression levels of caspase 3, PARP, p-Akt, p-p38, p-Erk 1/2, p-Smad2 and p-Smad3 in HOS cells treated with CUR, DMC and BDMC. The PARP cleavage has long been considered as a b typical biochemical hallmark of apoptosis. PARP was cleaved by caspase 3 during apoptosis in human osteosarcoma cells. The level of cleaved PARP and cleaved caspase 3 were increased on higher-dose (20 μM) CUR, DMC and BDMC treatment in HOS cells, but low dose (10 μM) did not (Fig. 5**a**). Additionally, the Smad, Akt, Erk and p38 signaling pathway also played important roles in inducing apoptosis of osteosarcoma cells. CUR, and DMC significantly induced the phosphorylation of p-Smad2 and p-Smad3 in HOS cells, but BDMC did not. BDMC decreased the phosphorylation of Akt in HOS cells, but it did not affect the phosphorylation of ERK and p38 (Fig. 5**b**).

### CUR, DMC and BDMC combination were evaluated the anticancer activity against the HOS cells

**Fig. 6:**
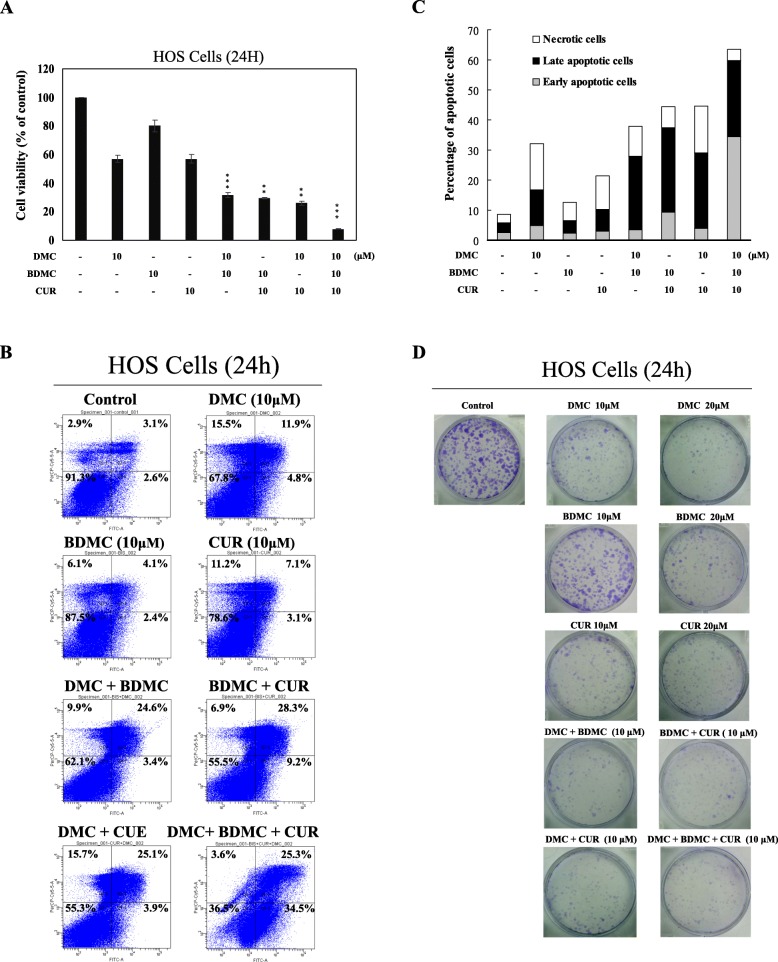
The combined effects of CUR, DMC, and BDMC on cell viability (**a**), apoptosis (**b**), and colony formation (**d**) in HOS cells**.** HOS cells treated with 10 μM CUR, DMC, and BDMC or their combination as indicated in the figure for 24 h were detected using MTT assay, annexin V-FITC /7-AAD staining, and clonogenic assay. **c** Quantitative analysis of the percentage of early apoptotic and late apoptotic cells in HOS cells. The percentage of cell viability was calculated as the ratio: 100% x (A540 absorbance of the drug-treated wells / (A540 absorbance of the no drug-treated wells). * Indicates the values are significantly different than the control (*P < 0.05, **P < 0.01, ***P < 0.001)

The anticancer effects of CUR, DMC and BDMC combination on HOS cells were evaluated using the MTT test of cell viability, flow cytometric analysis of cellular DNA content, and colony formation analysis of cell proliferation. First, the combination of three compounds (CUR, DMC and BDMC) reduced cell viability better than either two or a single agent in HOS cells (Fig. 6**a**). The combination of the three compounds also synergistically increased apoptosis (Fig. 6**b** and **c**). Overall, the combination of the three compounds could be used as a novel target for the treatment of osteosarcoma. We next performed a colony formation assay to evaluate the effects of combinations of CUR, DMC and BDMC on HOS cell proliferation. The low dose (10 μM) CUR and DMC treatment in HOS cells reduced the colony formation, but the low dose (10 μM) BDMC did not. The higher dose (20 μM) CUR, DMC, and DBMC significantly reduced the colony formation. The combined 10 μM BDMC with 10 μM CUR or DMC synergistically reduced the colony formation than single agents alone in HOS cells (Fig. 6**d**).

## Discussion

The pharmacological activity of turmeric has been attributed mainly to the three curcuminoid constituents, including CUR, DMC and BDMC. Although CUR was well known for its anti-tumor, anti-oxidant, anti-inflammatory, anti-neurotoxicity, anti-angiogenesis, anti-viral, and anti-bacterial, the ability of two related compounds DMC and BDMC in turmeric were less well known. The present study found that all three curcuminoids (CUR, DMC and BDMC) are equipotent in inducing HOS cell apoptosis by caspase-dependent and -independent pathways through activation of Smad2/3 and repression of Akt signaling pathway. Furthermore, we demonstrated that the combination of the three curcuminoids synergistically increased apoptosis than either two or a single agent in HOS cells. The synergistic effects of these three curcuminoids were in agreement with the previously reported studies which indicated that the curcuminoid mixture was more active than the individual components [1]**.**

Previous studies reported that the relative potency for decreasing of TNF-α induced NF-κB activation was Cur > DMC > BDMC [1]. Our findings were similar to previous study. Cur and DMC reduced cell viability better than BDMC in MDA-MB-231 and A2058 cancer cells. BDMC was chemically more stable than CUR and DMC, but its anti-breast cancer or anti-melanoma effects were lower than CUR and DMC. However, equimolar concentrations of CUR, DMC and BDMC had the same inhibitory effect on HOS and U2OS cells. CUR and DMC contain either two or one phenyl methoxy groups, whereas BDMC contains none. These results suggested that the methoxy groups on the phenyl ring played a more important role in the prevention of breast and melanoma cancer in comparison to osteosarcoma. While it is unclear why the different cell lines exhibited distinct sensitivity to suppression of cellular proliferation after CUR, DMC, or BDMC treatment.

Many research had shown that three curcuminoids (CUR, DMC and BDMC) are hypomethylation agent to suppress DNA methyltransferases (DnMT) activity in non–small cell lung cancer cell lines [24]. BDMC exhibited much better chemical stability, demethylation effect, and anti-invasion ability [14] than CUR and DMC. In this study, CUR, DMC and BDMC have the same inhibitory effect on HOS and U2OS cells under the same concentrations. Surprisingly, we observed that CUR and DMC induced the apoptosis of HOS cells through activating of Smad2/3 signaling pathway, but BDMC did not. A recent study has found that the overexpression of p-Smad2, p-Smad3, and Smad4 increased caspase 3 and caspase 9 activation in osteosarcoma cells [25]. Notably, we found that CUR, and DMC significantly induced caspase 3 activation and p-Smad2/3 phosphorylation in HOS cells. These results suggested that CUR, and DMC induced caspase-mediated apoptosis in HOS cells through the Smad signaling pathways. Additionally, the low expression of inhibitor of growth family member 5 (ING5) which was a tumor suppressor gene was detectable in HOS cells. Overexpression of ING5 in HOS cells induced apoptosis by activating the Smad2/3 and caspase 3/9 [25]. Thus, whether CUR and DMC also could regulate ING5 protein expression remain to be investigated. However, BDMC induced caspase-mediated apoptosis in HOS cells through the Akt signaling pathways. Activation of Smad2/3 or inhibition of Akt may represent a promising approach for the targeted therapy for osteosarcoma. Because many signaling pathways played vital roles in the development of cancer, using targeted agents to inhibit multiple signaling pathways are frequently deregulated in cancers [26–28]. This suggested that collective action rather than just a single effect became more important for anticancer therapy. Thus, synergistic induction of apoptosis by combination treatment with these three compounds might regulate multiple signaling pathways in osteosarcoma. Further study will be required to investigate the molecular mechanisms of combined CUR, DMC and BDMC and evaluate the anticancer effects of three compounds on murine xenograft models of human osteosarcoma cell lines. Overall, these findings provide novel insight into the combination of these three compounds.

## Conclusions

In the present study, CUR,DMC, and BDMC all decreased cell viability in osteosarcoma (HOS and U2OS), breast (MDA-MB-231), and melanoma (A2058). Additionally, CUR,DMC, and BDMC all decreased cell viability and induced the apoptosis of HOS cells through activation of Smad 2/3 and repression of Akt signaling pathway. Furthermore, we also want to examine the combined effects of CUR, DMC and BDMC on HOS cell proliferation. The combination of CUR,DMC, and BDMC synergistically reduced the cell viability, colony formation and increased apoptosis than either two or a single agent in HOS cells. Overall, the combination of these three compounds could be used as a novel target for the treatment of osteosarcoma.

## Supplementary information

**Additional file 1.**

## Data Availability

The datasets used and/or analysed during the current study are available from the corresponding author on reasonable request.

## Abbreviations

AIF: Apoptosis-inducing factor
BCRC: Bioresource Collection and Research Center
BDMC: Bisdemethoxycurcumin
CUR: Curcumin
DMC: Demethoxycurcumin
DMEM: Dulbecco’s Modified Eagle’s Medium
ECL: Chemiluminescence
ING5: Inhibitor of growth family member
PBS: Phosphate buffered saline
