# Supplementary figures and images for: Curcumin, demethoxycurcumin, and bisdemethoxycurcumin induced caspase-dependent and –independent apoptosis via Smad or Akt signaling pathways in HOS cells
Source: BMC Complement Med Ther. 2020 Mar 3;20:68. doi: 10.1186/s12906-020-2857-1 (PMC7076840; doi:10.1186/s12906-020-2857-1)

## Slide 1
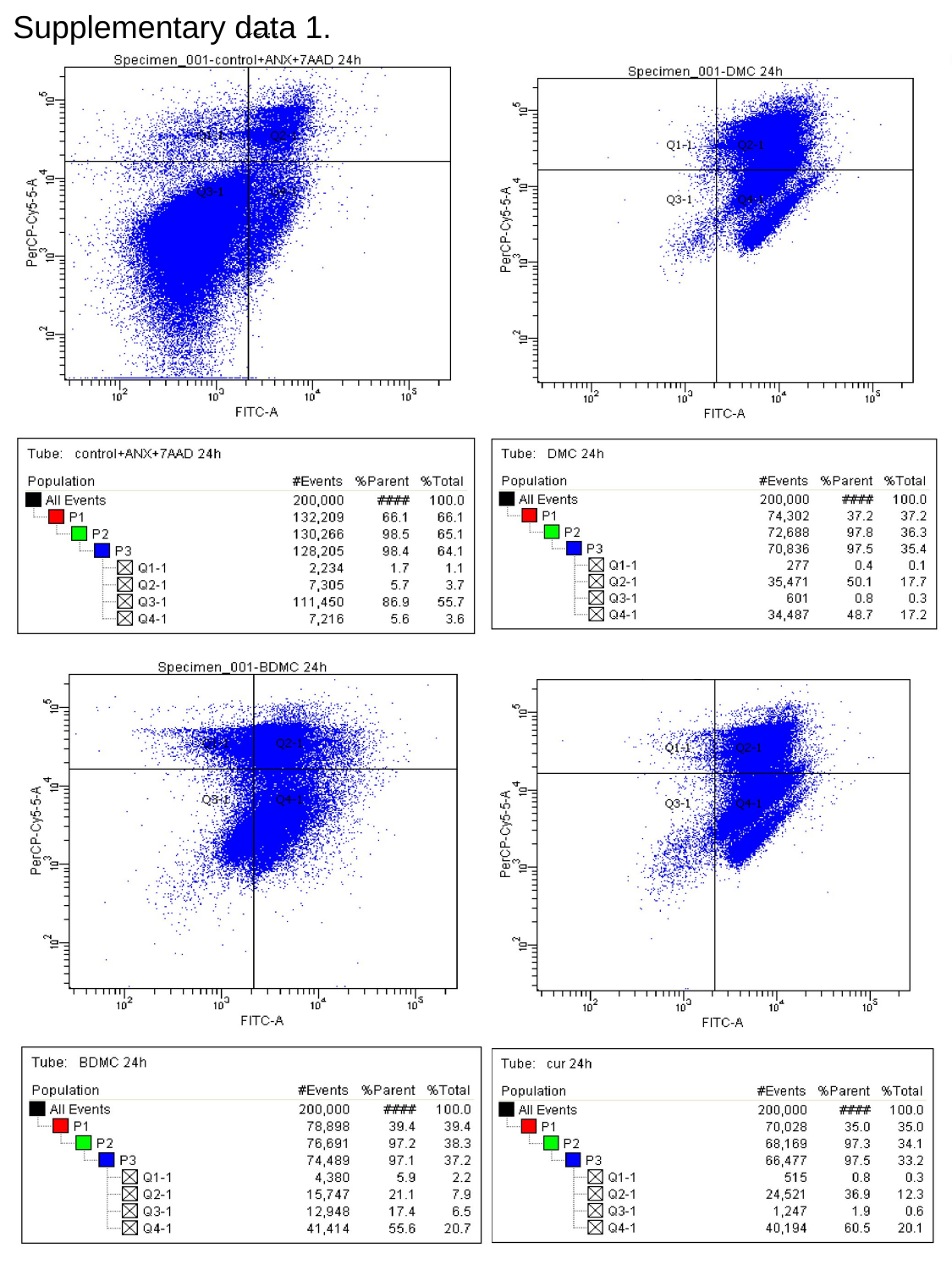

supplementary data
Supplementary data 1.

## Slide 2
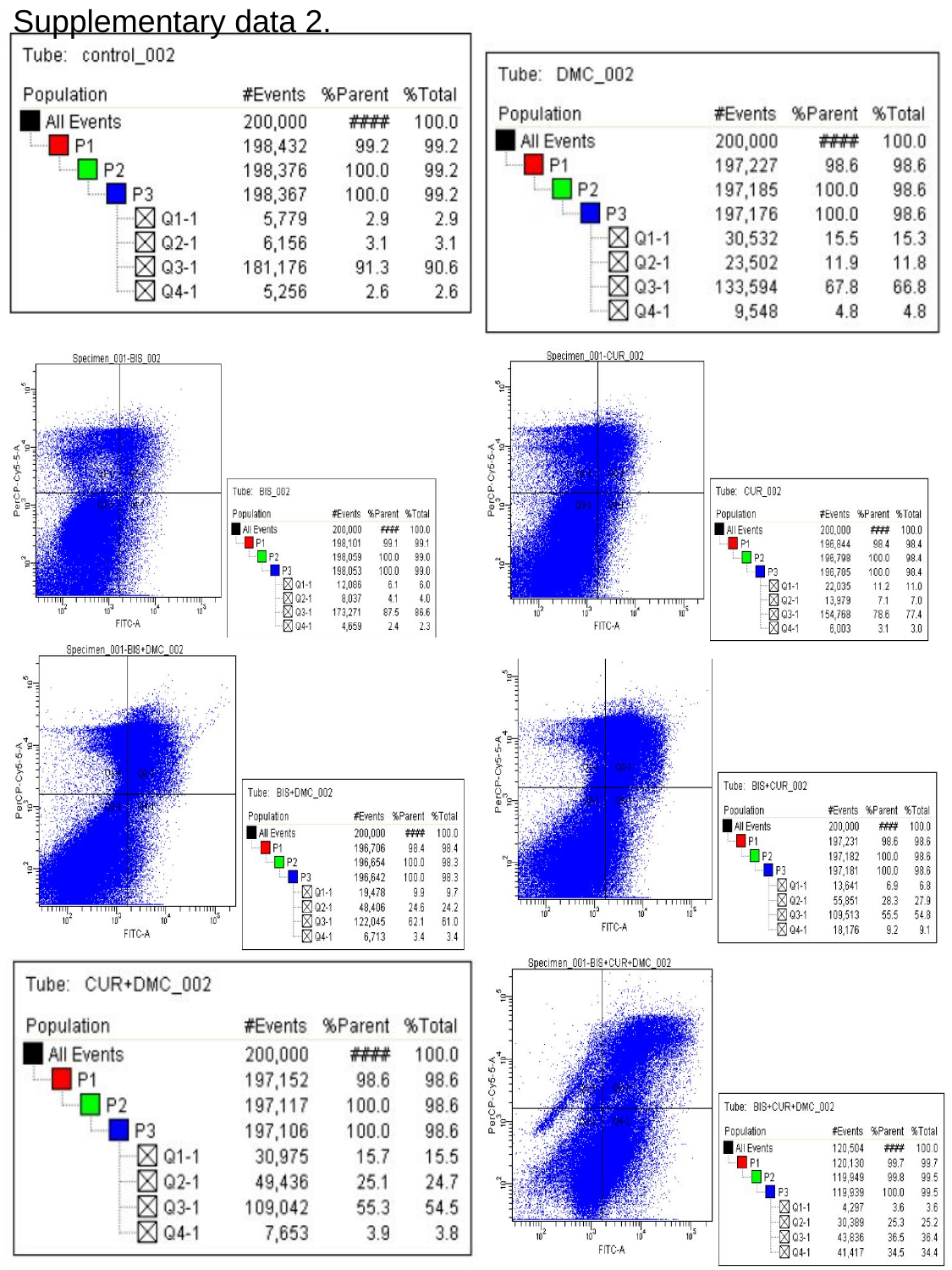

Supplementary data 2.

Supplement: Supplementary file 1 — Additional file 1. [file 12906_2020_2857_MOESM1_ESM.ppt]
